# Meta‐analysis of non‐linear exposure‐outcome relationships using individual participant data: A comparison of two methods

**DOI:** 10.1002/sim.7974

**Published:** 2018-10-03

**Authors:** Ian R. White, Stephen Kaptoge, Patrick Royston, Willi Sauerbrei, The Emerging Risk Factors Collaboration

**Affiliations:** ^1^ MRC Clinical Trials Unit University College London London UK; ^2^ Department of Public Health and Primary Care University of Cambridge Cambridge UK; ^3^ Faculty of Medicine and Medical Center – University of Freiburg Germany; ^4^ ERFC Investigators/Collaborators are listed in the Appendix (Supplementary Materials)

**Keywords:** fractional polynomials, meta‐analysis, multivariate meta‐analysis, prognostic research, random effects models

## Abstract

Non‐linear exposure‐outcome relationships such as between body mass index (BMI) and mortality are common. They are best explored as continuous functions using individual participant data from multiple studies. We explore two two‐stage methods for meta‐analysis of such relationships, where the confounder‐adjusted relationship is first estimated in a non‐linear regression model in each study, then combined across studies. The “metacurve” approach combines the estimated curves using multiple meta‐analyses of the relative effect between a given exposure level and a reference level. The “mvmeta” approach combines the estimated model parameters in a single multivariate meta‐analysis. Both methods allow the exposure‐outcome relationship to differ across studies. Using theoretical arguments, we show that the methods differ most when covariate distributions differ across studies; using simulated data, we show that mvmeta gains precision but metacurve is more robust to model mis‐specification. We then compare the two methods using data from the Emerging Risk Factors Collaboration on BMI, coronary heart disease events, and all‐cause mortality (>80 cohorts, >18 000 events). For each outcome, we model BMI using fractional polynomials of degree 2 in each study, with adjustment for confounders. For metacurve, the powers defining the fractional polynomials may be study‐specific or common across studies. For coronary heart disease, metacurve with common powers and mvmeta correctly identify a small increase in risk in the lowest levels of BMI, but metacurve with study‐specific powers does not. For all‐cause mortality, all methods identify a steep U‐shape. The metacurve and mvmeta methods perform well in combining complex exposure‐disease relationships across studies.

## 1 INTRODUCTION

It is often of primary interest in epidemiological studies to assess the nature of the relationship between continuous exposure(s) or covariate(s) and disease outcome(s), with or without adjustment for confounders. Moreover, with increasing adoption of collaborative research (or consortia approaches) in many disease fields to help standardize methodology and improve statistical power by meta‐analysis,^1^, ^2^ there is increasing interest in making such assessments, especially in the context of meta‐analysis of individual participant data (IPD) from multiple studies. Outcome data are often time to event in prospective studies or case‐control status in case‐control studies.

Methods for fitting non‐linear exposure‐outcome relationships are well established for the analysis of data from a single study. A commonly used approach is to categorize the exposure in model fitting, but this is sensitive to the choice of categories and risks losing power and information and introducing bias.^3^, ^4^ A better alternative, widely used and our main choice here, is fractional polynomials (FPs).^5^ Alternatives to FPs, which we discuss in Section 7.4, include ordinary polynomials,^6^ restricted cubic splines,^6^, ^7^ and other splines.^8^, ^9^

Two extensions to the meta‐analysis context, such as application to IPD meta‐analysis involving hundreds of studies and millions of participants, have been proposed: the metacurve approach 10 uses pointwise averaging of best‐fitting study‐specific functions, while the mvmeta approach 11, 12 uses multivariate meta‐analysis of study‐specific regression coefficients. These have not been broadly assessed for validity of results or to reveal any computational challenges.

Our broad aim is to compare the approaches proposed for the meta‐analysis of the shape of the relationship between a continuous exposure and an outcome, adjusted for one or more confounders using IPD from multiple studies. We specifically consider the Cox proportional hazards regression framework and two‐stage approaches, where the exposure‐outcome relationship is first estimated in each study and then combined across studies. The key question is how to combine relationships across studies. The aims of this paper are to evaluate and compare the metacurve and mvmeta approaches and hence to gain insight into when both methods, one method or neither method may be appropriate.

In Section 2, we describe the motivating data, a large IPD dataset from the Emerging Risk Factors Collaboration (ERFC), assessing the exposure‐outcome relationship of body mass index (BMI) with risk of coronary heart disease (CHD) and all‐cause mortality (ACM). In Section 3, we provide detailed descriptions of the two methods compared. We then evaluate the methods using mathematical comparison (Section 4), analysis of hypothetical data (Section 5), and analysis of the ERFC data (Section 6). In Section 7, we discuss the results, outline some advantages and disadvantages of each method, discuss some issues that need further investigation, and draw tentative conclusions.

## 2 DATA

The ERFC^13^ has requested and harmonized data from >120 prospective cohort studies in Western populations in order to facilitate IPD meta‐analysis of the associations of risk factors with incident cardiovascular disease outcomes and cause‐specific mortality. For the current analysis we use two ERFC datasets. Dataset 1 comprises 121 prospective cohorts and a total of 1 234 354 participants with data on BMI (exposure variable); age, sex and smoking status (confounders); and incident outcomes (55 780 CHD events; 225 356 ACM). The cohorts were based in Western Europe (82 cohorts, 734 779 participants), North America (25 cohorts, 417 726 participants), and other regions (Australia, Caribbean, Israel, Japan, and Turkey: 14 cohorts, 81 849 participants). In further modeling that might be used to explore the mechanism of association, we also adjust for systolic blood pressure (SBP), diabetes status, total cholesterol, and high‐density lipoprotein cholesterol (HDL‐C) as potential mediators using complete‐case data from 454 415 participants in 82 cohorts (dataset 2). For more details see other works.^13^, ^14^, ^15^, ^16^

To reduce the influence of extreme observations in modeling of continuous variables, we truncate the distribution of each continuous variable except age at its 1^st^ and 99^th^ centiles across cohorts, replacing values more extreme than these centiles by the values of these centiles.

## 3 METHODS

### 3.1 Notation

Denoting studies as i = 1, …, I, individuals as j = 1, …, J_i_, the continuous exposure of interest (BMI at baseline) as x_ij_, and confounders as a vector **c**_ij_ (possibly including non‐linear terms or interactions), the Cox proportional hazards model^17^ in study i may be written as:

(1) $$\log\;\left(h_{\mathrm{ij}}\left(t\right)\right)=\log\left(h_{0i}\;\left(t\right)\right)+f_{i}\;\left(x_{\mathrm{ij}};\beta_{i}\right)+\gamma_{i}^{\prime }c_{\mathrm{ij}},$$

where h_0i_(t) is a baseline hazard, f_i_(.) is defined below and **β**_i_ and **γ**_i_ are vectors of study‐specific coefficients. The parameters **β**_i_ and **γ**_i_ are estimated by maximising the partial likelihood for the data (time to event or censoring and event indicator).

### 3.2 Fractional polynomials in a single data set

FPs model the effect of an exposure x as $f\left(x;\beta \right)=\sum_{k=1}^{m}\beta_{k}x^{\;p_{k}}$ where m is the degree of the FP, the powers p_k_ are chosen from the set −2, − 1, − 0.5, 0, 0.5, 1, 2, 3, and **β** = (β_1_, β_2_, …, β_m_). In FP models, x^0^ is defined as log(x), and modifications allow two powers to be equal: for example, an FP model with degree 2 and powers (p, p) is defined as β_1_x^p^ + β_2_x^p^ log (x).^18^ The degree is constrained to be less than or equal to a limit called the dimension. Usually the dimension is set to 2 so that the degree is 1 or 2, denoted FP1 or FP2. Models of all degrees considered and all possible powers are fitted by maximising the likelihood or partial likelihood. The degree may be selected by a closed testing procedure^19^ or on subject‐matter grounds. The best‐fitting powers of the model with the selected degree are used.

### 3.3 Overall modeling strategy with fractional polynomials

The overall modeling strategy comprises five steps:

1. Select the confounders using subject matter knowledge and the data.
2. Select the degree of the FP function. This selection should not be performed within studies, which causes bias towards simple functional forms (here, towards linearity) because single studies tend to lack power to select complex functional forms.^5^, ^10^ Selection may be performed across studies by summing maximized likelihoods for each degree across studies or on subject‐matter grounds.
3. Select the powers in the FP function. This could be done either using study‐specific powers, giving
  (2) $$f_{i}\;\left(x;\beta_{i}\right)=\sum_{k=1}^{m}\beta_{\mathrm{ik}}x^{\;p_{\mathrm{ik}}},$$
  where **β**_i_ = (β_i1_, β_i2_, …, β_im_), or using common powers, giving
  (3) $$f_{i}\;\left(x;\beta_{i}\right)=f\;\left(x;\beta_{i}\right)=\sum_{k=1}^{m}\beta_{\mathrm{ik}}x^{\;p_{k}}$$
  (with the modifications noted above if powers equal each other or zero).
4. Fit the selected model to each study to get estimates ${\hat{\beta }}_{i}=\left({\hat{\beta }}_{i1},{\hat{\beta }}_{i2},\text{⋯},{\hat{\beta }}_{\mathrm{im}}\right)$ with variance‐covariance matrix **S**_i_.
5. Combine estimates or fitted functions across studies using the metacurve method (Section 3.4) or the mvmeta method (Section 3.5). Both methods involve meta‐analysis. The meta‐analysis may assume no heterogeneity: this is usually called the fixed‐effect model, but we follow Higgins et al^20^ in calling it the common‐effect (CE) model. Alternatively the meta‐analysis may allow for heterogeneity using the random‐effects (RE) model. With the RE model, the empirical Bayes estimates of the study‐specific curves may help in understanding the nature of the heterogeneity.^21^

### 3.4 Pooling method 1: Metacurve

The metacurve approach (implemented in the Stata package metacurve) conducts pointwise averaging of estimated study‐specific curves, and proceeds as follows:

1. Choose reference level x_0_ (eg, the overall mean of x).
2. For each x in the data (pointwise):
  - For each study i, estimate ${\hat{f}}_{i}\;\left(x,x_{0}\right)=f_{i}\;\left(x;{\hat{\beta }}_{i}\right)-f_{i}\;\left(x_{0};{\hat{\beta }}_{i}\right)$ and derive its standard error from **S**_i_.
  - Perform meta‐analysis (CE or RE) on the ${\hat{f}}_{i}\left(x,x_{0}\right)$ to get a pooled estimate $\hat{f}\;\left(x,x_{0}\right)$ and its variance.
3. Graph $\hat{f}\;\left(x,x_{0}\right)$ against x. Add pointwise confidence bands.

The metacurve approach is a direct way to pool estimated curves. However, different choices of reference level may give different answers. More important is the variation of study weights which reflect at each point the information in each of the studies, relevant if the distribution of x varies across studies. The next proposal avoids these issues.

### 3.5 Pooling method 2: Mvmeta

The mvmeta (multivariate meta‐analysis) approach uses meta‐analysis of the set of regression coefficients. This only works when common powers have been used across studies, as in Equation (3). The approach proceeds as follows.

1. Combine the ${\hat{\beta }}_{i}$ and **S**_i_ using multivariate meta‐analysis (CE or RE) to give an overall mean $\hat{\beta }$ and its variance **S**.
2. Draw fitted curves of $\hat{f}\;\left(x,x_{0}\right)=f\;\left(x;\hat{\beta }\right)-f\;\left(x_{0};\hat{\beta }\right)$ where the reference level x_0_ is only used to graph the estimated curves. Add pointwise confidence bands.

### 3.6 Categorized exposure

An alternative to FPs, included here because it is frequently used in the epidemiological literature, is to categorize the exposure into K groups and fit K − 1 dummy variables. For example, let I_k_(x) be an indicator of x falling in the kth group, and take the rth group as the reference group. This strategy fits into the framework presented in Section 3.1 above with $f_{i}\left(x;\beta_{i}\right)=\sum_{{k=1}_{k\neq r}}^{K}\beta_{\mathrm{ik}}I_{k}\left(x\right)$ and **β**_i_ = (β_i1_, …, β_ir − 1_, β_ir + 1_, …, β_iK_). The first stage of estimation involves fitting model (1) to each study separately, yielding estimates ${\hat{\beta }}_{i}$ with variance‐covariance matrix **S**_i_. Whether we choose the CE or RE model, we must also choose to perform the meta‐analysis separately for each ${\hat{\beta }}_{\mathrm{ik}}$ (univariate meta‐analysis), or jointly for the whole vector ${\hat{\beta }}_{i}$ (multivariate meta‐analysis).

### 3.7 Approach to method comparison

We evaluate the metacurve and mvmeta methods in three ways. First, we use mathematics to identify when they are likely to agree and when they are likely to differ, in the common powers case (Section 4). Second, we create hypothetical data sets to explore the extent of differences in rather extreme cases with common powers (Section 5). Third, we analyze the ERFC data using metacurve and mvmeta, and investigate any differences (Section 6). In this comparison, we include metacurve with study‐specific powers as well as with common powers, because the study‐specific powers option is a potential advantage of metacurve, affording greater flexibility. We also perform a sensitivity analysis of the ERFC data using metacurve in order to assess the importance of choice of reference level.

### 3.8 Summary of FP pooling methods

**eTable 1** summarizes the modeling approaches we have described and (in bold) options within those approaches: metacurve with study‐specific or common powers, mvmeta with common powers, and categorization. All approaches have the option of CE or RE meta‐analysis. Metacurve approaches further require a choice of reference level, while categorization approaches require a choice of number of categories and whether to perform univariate or multivariate meta‐analysis.

## 4 MATHEMATICAL EXPLORATION

We perform a mathematical comparison of metacurve and mvmeta, using the within‐studies model defined by Equations (1) and (3). For simplicity, we assume a correctly specified FP2 model with common powers, although this is a critical assumption in practice. Let the study‐specific parameters be **β**_i_ = (β_i1_, β_i2_) and the overall mean parameters be **β** = (β_1_, β_2_). The between‐studies model for CE meta‐analysis is **β**_i_ = **β**, and for RE meta‐analysis is **β**_i_∼N(**β**, **Σ**). For given reference level x_0_, our aim is to estimate the average log hazard ratio at exposure x compared to reference:

$$f\;\left(x,x_{0}\right)=\beta_{1}\;\left(x^{\;p_{1}}-x_{0}^{\;p_{1}}\right)+\beta_{2}\;\left(x^{\;p_{2}}-x_{0}^{\;p_{2}}\right)=a{\left(x\right)}^{T}\beta ,$$

where $a\left(x\right)={\left(x^{\;p_{1}}-x_{0}^{\;p_{1}},x^{\;p_{2}}-x_{0}^{\;p_{2}}\right)}^{T}$.

Our data yield estimates ${\hat{\beta }}_{i}∼N\left(\beta_{i},S_{i}\right)$ where **S**_i_ is the within‐study variance‐covariance matrix. We can write the marginal model as ${\hat{\beta }}_{i}∼N\left(\beta ,V_{i}\right)$ where **V**_i_ = **S**_i_ for the CE meta‐analysis and $V_{i}=S_{i}+\hat{\Sigma }$ for the RE meta‐analysis, and the heterogeneity variance matrix $\hat{\Sigma }$ is estimated from the data.

The mvmeta approach uses the multivariate pooled estimate $\hat{\beta }=\sum_{i}W_{i}{\hat{\beta }}_{i}$ where $W_{i}={\left(\sum_{i}V_{i}^{-1}\right)}^{-1}V_{i}^{-1}$, giving an estimate of f(x, x_0_) given by

$${\hat{f}}_{mv}\left(x,x_{0}\right)=a{\left(x\right)}^{T}\hat{\beta }=\sum_{i}a{\left(x\right)}^{T}W_{i}{\hat{\beta }}_{i}.$$

The metacurve approach on the other hand pools univariate estimates $a{\left(x\right)}^{T}{\hat{\beta }}_{i}$ with their variances v_i_(x), where v_i_(x) = **a**(x)^T^**S**_i_**a**(x) for the CE meta‐analysis and $v_{i}\left(x\right)=a{\left(x\right)}^{T}S_{i}a\left(x\right)+\hat{\sigma }{\left(x\right)}^{2}$ for the RE meta‐analysis, and $\hat{\sigma }{\left(x\right)}^{2}$ is estimated from the data. Then, the pooled estimate is

$${\hat{f}}_{\mathrm{mc}}\left(x,x_{0}\right)=\sum_{i}w_{i}\left(x\right)a{\left(x\right)}^{T}{\hat{\beta }}_{i}$$

with weights w_i_(x) = v_i_(x)^−1^/∑_i_v_i_(x)^−1^.

We consider two special cases. First, suppose all studies have a similar distribution of covariates. In linear regression on covariates $x_{\mathrm{ij}}^{\;p_{1}},x_{\mathrm{ij}}^{\;p_{2}},c_{\mathrm{ij}}$, the variance‐covariance matrix **S**_i_ of the coefficients is proportional to the inverse variance‐covariance matrix of the covariates. In non‐linear regressions such as Cox regression, the same statement is true for a weighted variance‐covariance matrix of the covariates, where the weights allow for individual risk. Hence, **S**_i_ is approximately proportional to the inverse variance‐covariance matrix of the covariates, the approximation being good for small coefficients and covariate‐independent censoring. If in addition all studies have a similar distribution of covariates, it follows that **S**_i_ ≈ λ_i_**S** is likely to hold for scalar λ_i_ and matrix **S**. Under CE meta‐analysis, this implies **V**_i_ ≈ λ_i_**V**, which we call “proportional variances.” Proportional variances occur in the RE model if additionally (i) all **S**_i_ are much larger than $\hat{\Sigma }$, (ii) all **S**_i_ are much smaller than $\hat{\Sigma }$ (so $V_{i}\approx \hat{\Sigma }$), or (iii) $S=\nu \hat{\Sigma }$ for some scalar ν.

The second special case arises if v_i_(x) = **a**(x)^T^**V**_i_**a**(x). This is always true under a CE meta‐analysis, but typically not true under a RE meta‐analysis because univariate and multivariate models yield different heterogeneity variance estimates.^22^ It occurs under a RE meta‐analysis if all **S**_i_ are much larger than **Σ**, so that we are close to the CE model, or if heterogeneity variance estimates are stable between univariate and multivariate models, which would occur if there were little borrowing of strength. We call this second case “stable heterogeneity.”

In the case of proportional variances, it follows that $W_{i}=w_{i}^{*}I$, where scalar $w_{i}^{*}={\left(\sum_{i}\lambda_{i}^{-1}\right)}^{-1}\lambda_{i}^{-1}$ and **I** is the 2 × 2 identity matrix. Then, ${\hat{f}}_{mv}\left(x,x_{0}\right)$ simplifies to $\sum_{i}w_{i}^{*}\;a{\left(x\right)}^{T}{\hat{\beta }}_{i}$ which is a weighted average of study‐specific x − x_0_ contrasts. If addition, we have stable heterogeneity, then $w_{i}\left(x\right)=w_{i}^{*}$ for all x, the weights are equal across the two methods and ${\hat{f}}_{mv}\left(x,x_{0}\right)={\hat{f}}_{\mathrm{mc}}\left(x,x_{0}\right)$.

If on the other hand proportional variances does not hold, then the mvmeta estimate is not a combination of study‐specific x − x_0_ contrasts: it therefore draws strength from other aspects of the fitted curve. We argue that mvmeta therefore borrows strength and gains precision if the model is correct but may lose robustness (that is, have greater error) if the model is mis‐specified. These results motivate the hypothetical data below.

## 5 HYPOTHETICAL DATA

We compare the metacurve and mvmeta methods using four hypothetical data sets. using studies with very different exposure distributions to explore the implications of non‐proportional variances. For simplicity, we use only two studies and consider only quadratic polynomials: that is, we use FP2 with common powers (1, 2). Our aims are: to compare the precisions of the two methods when both studies follow the same quadratic polynomial (a correctly specified model, example 1); to compare the methods’ ability to approximate the true exposure‐outcome relationship when both studies follow the same curve that is not a quadratic polynomial (a mis‐specified model, examples 2 and 3); and to explore possible artefacts due to weights varying with exposure (example 4).

In examples 1‐3, the studies have adjacent but non‐overlapping ranges of the exposure x (BMI): uniformly distributed between 20 and 25 in Study 1 and between 25 and 30 in Study 2. The two studies are both large (size 500 000) to minimize noise. A time‐to‐event outcome is simulated from a proportional hazards model. We fit quadratic models followed by CE meta‐analyses, with reference point x_0_ = 25 for metacurve.

**Figure 1:**
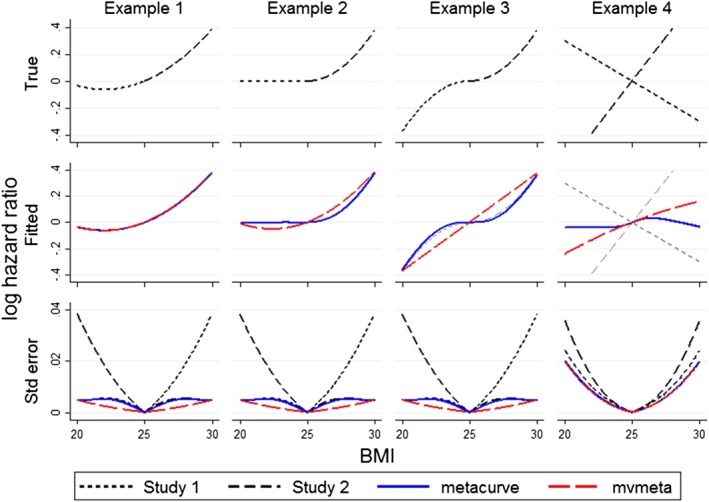
Comparison of metacurve and mvmeta methods: Simulated data. BMI, body mass index [Colour figure can be viewed at wileyonlinelibrary.com]

In Example 1, the true model is quadratic with minimum at x = 22 (Figure 1, top left panel). Both mvmeta and metacurve correctly reproduce this curve (Figure 1, middle left panel), but metacurve has larger standard error than mvmeta (Figure 1, bottom left panel). Note that, by definition, standard errors are zero at the reference point x = 25. The difference in standard errors arises because at x < 25 metacurve yields standard errors approximately equal to those for Study 1 (leading the two curves almost to coincide), indicating that it draws very little information from Study 2 in this range, and similarly at x > 25 metacurve draws information primarily from Study 2. The mvmeta method, on the other hand, can draw information from both curves.

In Example 2, the true model is flat at *x* < 25 and a quadratic at *x* > 25 (Figure 1 second column). This is mis‐specified because it is not a quadratic model. The fitted curves are wrong for both mvmeta and metacurve, but metacurve is much closer to the truth. Differences in standard errors are similar to those in Example 1. In Example 3, the true model follows different quadratics at *x* < 25 and *x* > 25 (Figure 1, third column). In this case, metacurve again approximates the true model well, but mvmeta (which is restricted to quadratics) misses all the curvature.

In Example 4, the studies have overlapping exposure ranges: BMI is uniformly distributed between 20 and 30 in study 1, and between 22 and 28 in study 2 (Figure 1, last column). The sample sizes are 25 000 and 75 000 respectively. Further, the two studies follow very different linear models. When they are pooled using metacurve, the impact of the weights varying with BMI becomes clear, as study 1 dominates for BMI around 25 (giving a positive gradient) while study 2 dominates for BMI below 22 and above 28 (giving a negative gradient). This demonstrates that weights varying with exposure can yield implausible conclusions: however, this is an extreme example where it is questionable whether the curves should be combined.

## 6 ANALYSIS OF ERFC DATA

### 6.1 Data summary

Table 1 summarizes the EFRC data used for modeling. Dataset 1 is used for the confounder‐adjusted analyses in Sections 6.2 to 6.4; dataset 2 is used for the analyses additionally adjusted for mediators in Section 6.5. Between‐cohort variation in risk factors was generally smaller than within‐cohort variation, except for age for which the within‐ and between‐cohort SDs were similar. Cohort‐specific distributions of age and BMI are shown in the supplementary materials (**eFigure 1**). These findings suggest that exposure distributions vary between studies and therefore the results in Section 4 suggest that metacurve and mvmeta approaches are likely to differ. We set the reference level *x*_0_ at 25 kg/m^2^ (close to the overall mean of just over 26 kg/m^2^) because this is the WHO common cutoff for overweight.^16^, ^23^ We choose to use FP2 functions on subject‐matter grounds, because previous studies suggest non‐monotonic relationships.^15^, ^16^ An FP1 function would be preferred if a monotonic function were expected.

**Table 1:** Summary of the Emerging Risk Factors Collaboration data used for modeling

Characteristic	Summary	Dataset 1			Dataset 2		
		(121 Cohorts, 1 234 354			(82 Cohorts, 454 415		
		Participants)			Participants)		
		Summary	Within	Between	Summary	Within	Between
			Cohort SD	Cohort SD		Cohort SD	Cohort SD
*Outcomes*							
CHD events	n(%)	55 780(5.72%)	‐	‐	17 947(5.14%)	‐	‐
All‐cause mortality	n (%)	225 356(23.0%)	‐	‐	59 771(20.2%)	‐	‐
First event follow up (years)	Median(5^th^, 95^th^*)	12.6 (3.7, 30.5)	‐	‐	11.0 (3.3, 22.5)	‐	‐
Mortality follow up (years)	Median (5^th^, 95^th^*)	13.7 (4.5, 30.8)	‐	‐	11.9 (4.0, 23.0)	‐	‐
*Covariates*							
BMI (kg/m^2^)	Mean	26.1	4.2	1.2	26.3	4.2	1.2
Age at baseline (years)	Mean	55.5	10.0	8.2	57.1	9.4	8.9
Sex (female)	n (%)	642 419(53.0%)	(39.0%)	(9.5%)	217 601(52.3%)	(42.3%)	(10.7%)
Smoking status (current)	n (%)	360 765(31.2%)	(43.4%)	(14.9%)	136 654(28.9%)	(43.9%)	(14.3%)
SBP (mmHg)	Mean	‐	‐	‐	136	19.0	8.0
Diabetes (yes)	n (%)	‐	‐	‐	26 203(6.6%)	(21.4%)	(3.3%)
Total cholesterol (mmol/l)	Mean	‐	‐	‐	5.83	1.1	0.5
HDL‐C (mmol/l)	Mean	‐	‐	‐	1.35	0.4	0.2

SD: standard deviation. CHD: coronary heart disease. BMI: body mass index. HDL‐C: high density lipoprotein cholesterol.

* 5^th^ and 95^th^ centiles

### 6.2 Main meta‐analysis results: BMI and CHD adjusted for confounders in dataset 1

**Figure 2:**
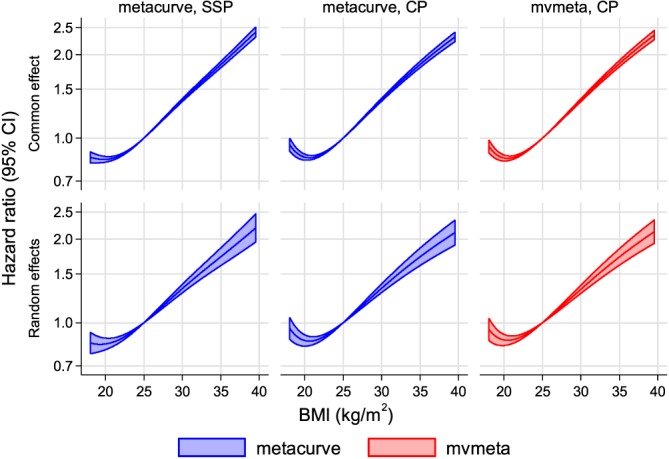
Meta‐analysis of BMI‐CHD association using FPs: Comparison of metacurve vs. mvmeta with study‐specific powers (SSP) or common powers (CP). Dataset 1, adjusted for confounders. With SSP, only metacurve can be used. BMI, body mass index; CHD, coronary heart disease; FP: fractional polynomial [Colour figure can be viewed at wileyonlinelibrary.com]

The metacurve method with study‐specific FP powers showed an increasing association with a leveling off at low values of BMI (Figure 2, left‐hand panels). For the metacurve and mvmeta methods with common FP powers, the powers (selected across studies) were (−2, −2), implying the model *f*(*x*; ***β***) = *β*_1_*x*^−2^ + *β*_2_ log (*x*)*x*^−2^. Using these common FP powers, metacurve and mvmeta gave similar results (Figure 2, middle and right‐hand panels): both methods identified a J‐shape with nadir at 20.5 kg/m^2^, and unlike the results with study‐specific powers, a small increase in risk at the lowest levels of BMI. With the CE approach, confidence intervals were narrow; with RE they were a little wider.

**Figure 3:**
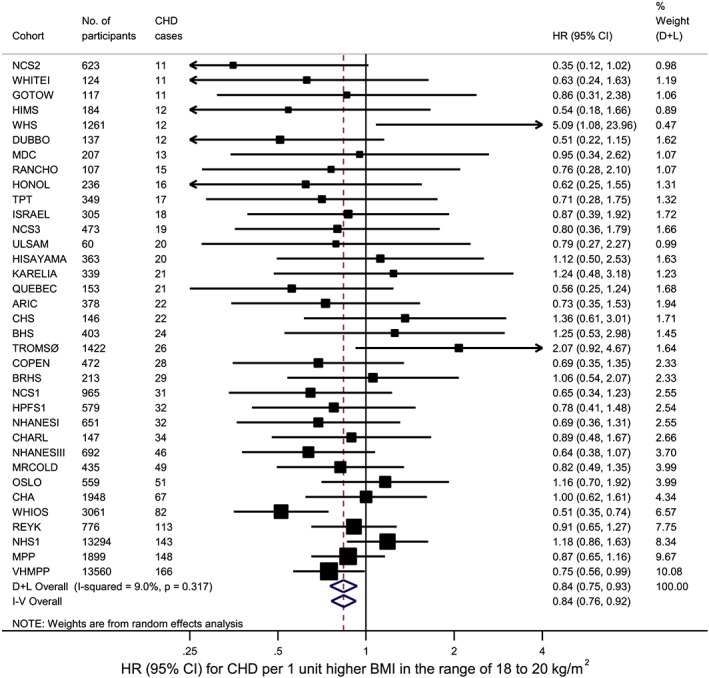
Random‐effects meta‐analysis of linear BMI‐CHD associations in the BMI range of 18 to 20 kg/m^2^. The meta‐analysis results are shown for 35 cohorts contributing >10 CHD cases within this BMI range. Dataset 1, adjusted for confounders. BMI, body mass index; CHD, coronary heart disease [Colour figure can be viewed at wileyonlinelibrary.com]

**Figure 4:**
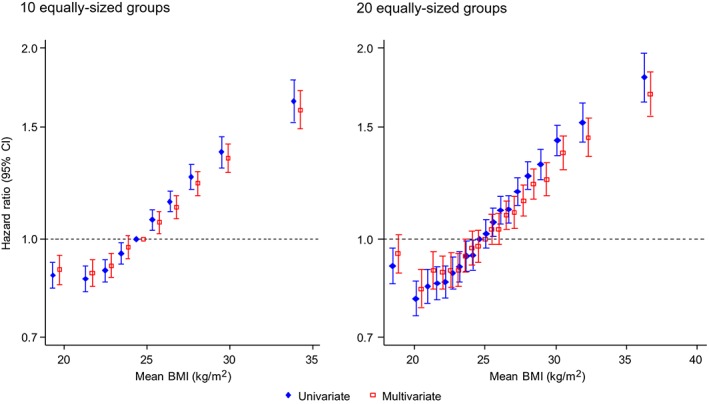
Random‐effects meta‐analysis of BMI‐CHD association using categorization approach. Dataset 1, adjusted for confounders. Overlapping data points are slightly offset in the x‐axis for better clarity. BMI, body mass index; CHD, coronary heart disease [Colour figure can be viewed at wileyonlinelibrary.com]

To explore whether the data truly support an upturn at low BMI, we conducted a sensitivity analysis by restricting data to the BMI range of 18 to 20 kg/m^2^, assuming the BMI‐CHD associations are linear in this short range, and performing a univariate meta‐analysis. This found a negative association (Figure 3) consistent with the upturn. We also used the exposure categorization method with categorization of BMI into 10 or 20 quantile groups across studies (Figure 4). The reference group in each case is the quantile group containing 25 kg/m^2^. With 10 groups, a plateau is apparent at the lowest end of the BMI distribution, but using 20 groups instead suggests a J‐shaped association. Inferences from univariate and multivariate meta‐analysis were broadly similar in these results. The analysis with 10 groups may not be sensitive enough to explore the upturn at low BMI, which is found by the analysis with 20 groups. Thus the upturn at low BMI appears real and the analyses with common FP powers appear to have performed better here than the analysis with study‐specific FP powers.

### 6.3 Illustration of the metacurve method

**Figure 5:**
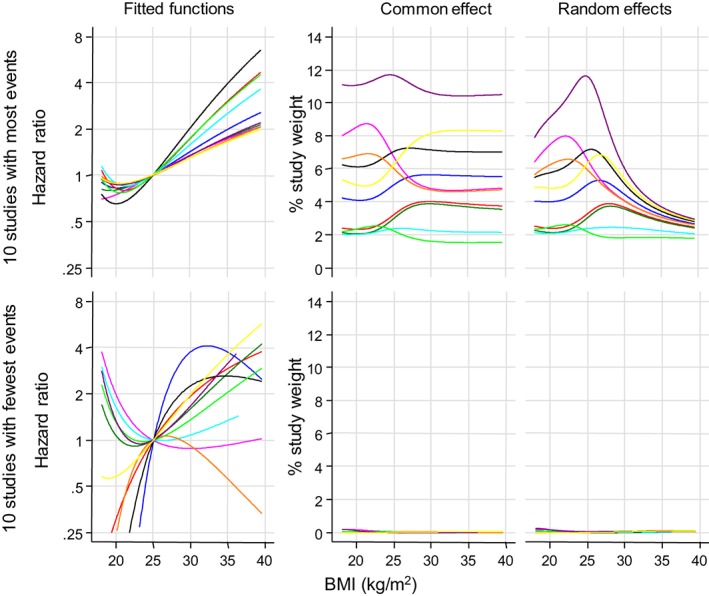
Fitted study‐specific functions for BMI‐CHD association in the 10 studies with most events (top: 1369 to 5829 events, mean = 3012) as compared to the 10 studies with fewest events (bottom: 11 to 26 events, mean = 17), and their weights in meta‐analysis. Dataset 1, adjusted for confounders. Studies are indicated by same line color within each of the top and bottom row. Fitted study‐specific hazard ratios are shown only above a lower limit of 0.25 for clarity. BMI, body mass index; CHD, coronary heart disease [Colour figure can be viewed at wileyonlinelibrary.com]

Figure 5 summarizes BMI‐CHD association results for 20 studies from the ERFC: in the top row, the 10 studies with most events (each contributing from 1369 to 5829 events, mean 3012); in the bottom row, the 10 studies with fewest events (contributing from 11 to 26 events, mean 17). The left‐hand panels show the fitted functions (with common FP powers). The middle and right‐hand panels graph the meta‐analysis percentage weight for each study against BMI level by meta‐analysis method (CE or RE). In the large studies, fitted study‐specific functions were consistent with the J‐shaped association evident in the overall meta‐analysis result. Study weights varied from 2% to 12% depending on BMI value and meta‐analysis method, with RE meta‐analysis shrinking study weights to be similar at the extremes of BMI levels (suggesting greater estimated between‐study heterogeneity). For the small studies, the fitted functions were noisy, and each study contributed <1% weight in any meta‐analysis. The empirical Bayes estimates revealed a greater extent of shrinkage in the functions of the smallest studies (**eFigure 2**).

### 6.4 Meta‐analysis of BMI and ACM adjusted for confounders in dataset 1

Meta‐analysis of the BMI‐ACM association using metacurve and mvmeta identified steep U‐shaped associations, irrespective of the method of FP power selection (**eFigure 3**), with nadirs for CE and RE meta‐analysis at 23.6 and 24.3 kg/m^2^ respectively. Using the categorization approach demonstrated clear U‐shaped associations whether using 10 groups or 20 groups (**eFigure 4**).

### 6.5 Meta‐analyses adjusted for confounders and mediators in dataset 2

We repeated the above analyses with further adjustment for mediators, thus estimating the direct effect of BMI on outcomes. Estimated overall curves were again very similar between FP methods for CHD (**eFigure 5**) and ACM (**eFigure 6**).

### 6.6 Choice of reference category

**Figure 6:**
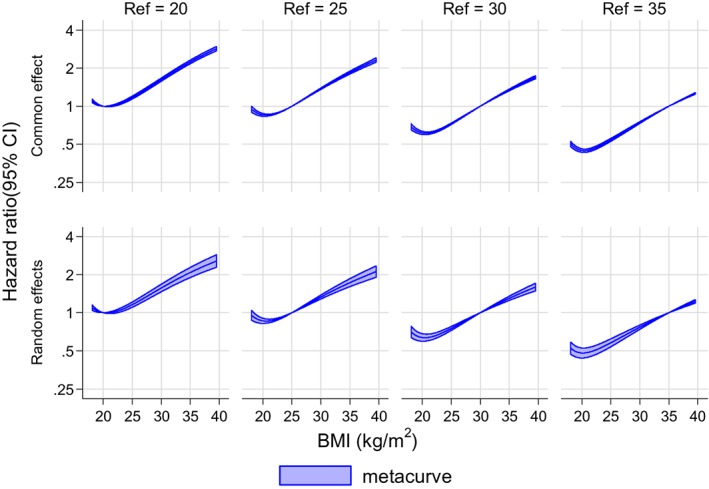
Comparison of metacurve results for BMI‐CHD association when varying the reference value of BMI (Ref) from 20 to 35 kg/m^2^. Dataset 1, adjusted for confounders. BMI, body mass index; CHD, coronary heart disease [Colour figure can be viewed at wileyonlinelibrary.com]

To explore whether metacurve results were sensitive to the choice of reference value *x*_0_, we varied this value to 20, 30, and 35 kg/m^2^. The fitted curves were similar in shape for BMI and CHD (Figure 6) and for BMI and ACM (**eFigure 7**).

## 7 DISCUSSION

### 7.1 Which method?

We have explored relationships between BMI and two outcomes using metacurve of FPs and mvmeta of FPs. Table 2 summarizes the advantages and disadvantages of the two methods. We now highlight the key points.

**Table 2:** Comparison of the fractional polynomial pooling methods

Characteristic	Metacurve	Mvmeta
Reference level	Required; has small effect on results	Not required
Use of fitted curves	Uses only local parts	Uses all of fitted curves (global)
Performance with correctly specified models	Less efficient	More efficient
Performance with incorrectly specified models	More robust	Less robust
Allows study‐specific FP powers	Yes	Not at present
Ease of use	Very easy: Metacurve in Stata	Easy: Mvmeta_make and mvmeta in Stata

Mathematical exploration showed that the two approaches tend to differ when exposure distributions vary considerably between studies, implying that mvmeta is more efficient when models are correctly specified and suggesting that metacurve is more robust to model mis‐specification. Hypothetical data simulated with extreme variation in exposure distributions between studies and rather extreme effects supported this view and showed that implausible pooled associations can in principle occur. In real analyses, exposure distributions always differ to some extent (see **eFigure 1** for an example) and the different weighting schemes should result in some differences between averaged curves. While mvmeta may have advantages because it borrows strength, metacurve may have advantages because the weights better reflect the information in the data. We found similar results for metacurve and mvmeta in the ERFC data, except that metacurve with study‐specific powers did not show an upturn in CHD risk at low levels of BMI. Closer exploration showed that these data did support the upturn, thus supporting the use of metacurve and mvmeta with common FP powers. In principle, the fit to the data should be better (or at least not worse) with study‐specific powers: metacurve with study‐specific powers may have performed worse because power selection in some studies was driven by other features of the data. Overall, these results suggest that the theoretical differences between methods may not be important in practice, and both methods may be recommended.

The results of metacurve are in principle sensitive to the choice of reference value. However, we did not find any important sensitivity over a wide range of reference values. In practice, subject‐matter knowledge often provides arguments for a suitable interval for the reference value, and then the specific choice made is highly unlikely to influence the result. Reference values should not be chosen outside the range of the data.

Both methods extrapolate study results beyond the range of their data. Metacurve extrapolates explicitly, since it averages weighted estimates from each study at each covariate value. Weights reflect the information at each point, implying that a study gets a relatively low weight in areas with a small number of participants (or events). Including studies only within their own covariate ranges is a possible alternative, but it risks artefacts in the curve shape where studies switch from included to excluded. Mvmeta extrapolates implicitly, by assuming the study‐specific curves are correct over the whole covariate range.

Exposure categorization may be useful as a first step to modeling when data are sufficient to accommodate fine categorization without much loss of precision, as in the current context of large‐scale IPD meta‐analysis. However, the results in Figure 4 highlight a major limitation of this approach, that the choice of categories affects the results and in particular the extent to which nonlinear associations may be apparent.

### 7.2 Outstanding issues

Outstanding issues in the metacurve and mvmeta methods include how to assess and compare model fit, which is complicated by the difficulty of pooling likelihood or discrimination over studies after random‐effects meta‐analysis; how to assess influential points; and how to display and interpret heterogeneity. Further, the impact of truncation (Section 2) is not fully understood. The methods proposed use IPD and are unlikely to be useful with aggregate data.

### 7.3 Confounders

Because of the considerable richness and standardization of the ERFC data, we were easily able to handle the common and difficult problem of adjusting for confounders. In much smaller meta‐analyses (say with about 5 to 10 studies), with larger differences between measured confounders, how to conduct a meta‐analysis adjusting for confounders is less straightforward. For a more detailed discussion, see Section 5 in Sauerbrei and Royston.^10^

### 7.4 Extensions

We have illustrated the methods using FPs as the method to derive functions in each study, but they apply for any model with a small number of parameters. For example, with restricted cubic splines,^24^ we would initially choose the number of knots (say *K*) and their location (eg, by model comparisons across all studies), then construct the appropriate spline basis (of size *K* − 1), and fit the model to each study separately. Metacurve would then pool the estimated relative effects exactly as with FPs, while mvmeta would pool the *K* − 1 coefficients. Results would depend on the choice of the knots.

For models with many parameters, eg, smoothing splines or penalized splines, metacurve would still apply as described. Here we propose implementing mvmeta by applying multivariate meta‐analysis to a set of relative effects estimated over a grid of exposure values. The size of the grid would need to balance accuracy with the computational difficulty of high‐dimensional multivariate meta‐analysis. This method is also appropriate to apply the mvmeta approach when study‐specific FPs have been selected.

In a randomized trial, it may be of interest to explore interactions between randomized treatment *z*_*i*_ and a continuous effect modifier *x*_*ij*_.^25^ The multivariable fractional polynomial interaction (MFPI) approach has been proposed to tackle this flexibly.^26^ To explore interactions across multiple trials, the linear predictor in Equation (1) is changed to *f*_*i*_(*x*_*ij*_; ***α***_*i*_) + *f*_*i*_(*x*_*ij*_; ***β***_*i*_)*z*_*ij*_, and the methods presented in this paper could be used to combine the treatment‐effect functions $f_{i}\left(x_{\mathrm{ij}};{\hat{\beta }}_{i}\right)$.^27^

The methods have been described for Cox models, but they can be applied to any model where the exposure is modeled through a linear predictor. Unlike the Cox model, such models usually include an intercept. It would therefore be possible to combine absolute risk curves (including the intercept) across studies; however, there is a danger of confounding by study. The methods presented for combining relative risk curves (ignoring the intercept) would be preferable since they only combine within‐study information.

### 7.5 Conclusions

The metacurve and mvmeta methods appear to perform well in combining complex exposure‐disease relationships across studies. Compared with the popular approach based on categorization, they use the full information from continuous variables, and results do not depend on the number and choice of cutpoints. Both methods rely on having good evidence from the study‐specific analyses, as was achieved here using a large consortium approach to standardize methodology and improve statistical power. Software is available in Stata for wider practical application.

## Supporting information

SIM7974_supplementary.pdf
