# Supplementary material for: Meta‐analysis of non‐linear exposure‐outcome relationships using individual participant data: A comparison of two methods
Source: Stat Med. 2018 Oct 3;38(3):326–38. doi: 10.1002/sim.7974 (PMC6492097; doi:10.1002/sim.7974)

SUPPLEMENTARY MATERIALS

APPENDIX: ERFC Investigators/Collaborators

AFTCAPS: Robert W Tipping; **ALLHAT:** Barry R Davis, Lara Simpson, Sara Pressel; **ARIC:** June Stevens, Chiadi Ndumele, David Couper; **AUSDIAB:** Dianna Magliano, Anna Peters, Jonathan Shaw; **BHS:** Matthew Knuiman, Mark Divitini; **BRHS:** Peter H Whincup, Goya S Wannamethee; **BRUN:** Stefan Kiechl, Johann Willeit, Peter Willeit; **BWHHS:** Juan-Pablo Casas, Caroline Dale, Debbie Lawlor; **CAPS:** John Gallacher, Yoav Ben-Shlomo; **CASTEL:** Edoardo Casiglia; **CHA:** Philip Greenland; **CHARL:** Paul J Nietert, Susan E Sutherland; **CHS:** Mary Cushman, Erika Enright; **CONOR (FINNMARK, HUBRO, OPPHED, OSLO2, TROMS):** Randi Selmer, Anne Johanne Søgaard, Lisa Lund Håheim; **COPEN:** Børge G Nordestgaard, Anne Tybjaerg-Hansen, Ruth Frikke-Schmidt; **CUORE (ATENA, MATISS83, MATISS87, MATISS93, MONFRI86, MONFRI89):** Simona Giampaoli, Luigi Palmieri, Diego Vanuzzo; **DRECE:** Miguel A Rubio-Herrera, Jose A Gutiérrez-Fuentes, A.Gómez de la Cámara; **DUBBO:** Leon A Simons; **EAS:** Jackie F Price, Stela McLachlan; **EPESEBOS:** James O Taylor; **EPESEIOW:** Robert B Wallace; **EPESENCA:** Gerda Fillenbaum, Dan G Blazer; **EPESENHA:** Caroline L Phillips; **EPICNOR:** Kay-Tee Khaw, Nicholas J Wareham; **ESTHER:** Ben Schöttker, Kai-Uwe Saum, Bernd Holleczek; **FINE_FIN:** Hanna Tolonen; **FINE_IT:** Chiara Donfrancesco, Simona Giampaoli; **FINRISK92:** Veikko Salomaa, Kennet Harald, Katja Borodulin; **FINRISK97:** Veikko Salomaa, Anne Juolevi, Antti Jula; **FUNAGATA:** Makoto Daimon, Toshihide Oizumi, Takeo Kato; **GOH:** Rachel Dankner, Angela Chetrit, Flora Lubin; **GOTO13:** Lars Wilhelmsen; **GOTO33:** Annika Rosengren, Lars Wilhelmsen, Lennart Welin; **GOTO43:** Annika Rosengren, Lars Wilhelmsen; **GOTOW:** Cecilia Björkelund, Lauren Lissner, Valter Sundh; **GRIPS:** Dorothea Nagel; **HBS:** Timo E Strandberg, Veikko Salomaa, Reijo S Tilvis; **HCS:** Cyrus Cooper, Elaine Dennison, Aran Aihie Sayer; **HELSINAG:** Reijo S Tilvis, Timo E Strandberg; **HIMS:** Leon Flicker, Bu Beng Yeap; **HISAYAMA:** Yutaka Kiyohara, Hisatomi Arima, Toshiharu Ninomiya; **HONOL:** Beatriz Rodriguez; **HOORN:** Coen DA Stehouwer, Jacqueline M Dekker, Giel Nijpels; **HPFS1:** Eric B Rimm, Walter C Willett, Alan Flint; **IKNS:** Hiroyasu Iso, Masahiko Kiyama, Akihiko Kitamura, Kazumasa Yamagishi; **ISRAEL:** Uri Goldbourt; **KARELIA:** Pekka Puska, Erkki Vartiainen, Tiina Laatikainen; **KIHD:** Jukka T Salonen, Jussi Kauhanen, Tomi-Pekka

Tuomainen; **LASA:** Marjolein Visser, Natasja van Schoor, Jan L Poppelaars; **LEADER:** Tom W Meade; **MCVDRFP:** WM Monique Verschuren; **MDC:** Olle Melander; **MESA:** Steven J Shea, Karol E Watson; **MONICA_KORA1:** Wolfgang Koenig, Christa Meisinger; **MONICA_KORA2:** Christa Meisinger, Wolfgang Koenig; **MONICA_KORA3:** Wolfgang Koenig, Christa Meisinger; **MORGEN:** WM Monique Verschuren; **MOSWEGOT:** Annika Rosengren; **MPP:** Bo Hedblad, Peter Nilsson, Olle Melander; **MRCOLD:** Astrid Fletcher; **MRFIT:** Lewis H Kuller; **NCS (NCS1, NCS2, NCS3):** Randi Selmer, Aage Tverdal; **NHANESI:** Hee-Choon Shin, Barbara Howard, Jason Umans; **NHANESIII:** Juan R Albertorio-Díaz, Hee-Choon Shin; **NHS1:** JoAnn E Manson, Frank B Hu, Eric B Rimm; **NPHSI:** Tom W Meade, Jackie A Cooper; **NPHSII:** Jackie A Cooper; **NSHS:** Jonathan A Shaffer, Karina W Davidson, Susan Kirkland; **OSAKA:** Shinichi Sato, Masahiko Kiyama, Akihiko Kitamura, Hiroyasu Iso; **OSLO:** Ingar Holme, Aage Tverdal; **OYABE:** Hidaeki Nakagawa, Masaru Sakurai; **PARIS1:** Pierre Ducimetiere; **PREVEND:** Stephan JL Bakker, Ron Gansevoort, Bruce Wolffenbuttel; **PRIME:** Philippe Amouyel, Jean Dallongeville; **PROCAM:** Gerd Assmann, Helmut Schulte; **PROSPER:** Naveed Sattar, J Wouter Jukema; **ProspectEPIC:** Yvonne T van der Schouw; **RANCHO:** Happy Araneta, Donna Kritz-Silverstein; **REYK:** Vilmundur Gudnason, Thor Aspelund, Bolli Thorsson; **RIFLE (ATS_SAR, GUBBIO, MICOL, MONICA, NFR, OB43, RF2):** Maurizio Trevisan; **RS_I:** Klodian Dhana, Maryam Kavousi, Oscar H Franco; **SHHEC:** Hugh Tunstall-Pedoe, Mark Woodward; **SHIP:** Henry Völzke, Carsten O Schmidt, Till Ittermann; **SHS:** Barbara V Howard, Lyle G Best, Momotaz Begum; **SPEED:** Yoav Ben-Shlomo, George Davey Smith; **TARFS:** Altan Onat, Gunay Can, Husniye Yuksel; **TOYAMA:** Hidaeki Nakagawa, Masaru Sakurai; **TPT:** Tom W Meade; **TROMSØ:** Inger Njølstad; **ULSAM:** Johan Sundström, Johan Ärnlöv, Ulf Risérus; **USPHS:** Michael J Gaziano, Paul M Ridker; **USRAIL:** David Jacobs, Henry Blackburn; **VHMPP:** Hanno Ulmer, Hans Concin, Gabriele Nagel; **VITA:** Francesco Rodeghiero; **WCWC:** Volker Arndt, Hermann Brenner, Dietrick Rothenbacher; **WHIOS:** Sylvia Wassertheil-Smoller; **WHITEI:** Mika Kivimaki, Michael G Marmot, Eric J Brunner; **WHITEII:** Eric J Brunner; **WHS:** Paul M Ridker; **WOSCOPS:** J Wouter Jukema, Ian Ford; **ZARAGOZA:** Alejandro Marín Ibañez; **ZUTE:** Daan Kromhout, Johanna M Geleijnse. We also acknowledge the use of data from the PRHHP and QUEBEC studies.

Data Management Team: Thomas Bolton, Sarah Spackman, and Matthew Walker.

Coordinating Centre: Thomas Bolton, Stephen Burgess, Adam S Butterworth, Emanuele Di Angelantonio, Stephen Kaptoge, Lisa Pennells, David Stevens, Sarah Spackman, Michael Sweeting, Simon G Thompson, Matthew Walker, Ian R White, Angela M Wood, and John Danesh (principal investigator).

Supplementary Tables and Figures

eTable 1. Summary of modelling approaches, showing options in bold. All methods have study-specific parameters. 5

eFigure 1. Box plots of distributions of age and BMI in 121 cohorts contributing to analyses. Dataset 1. BMI data are shown with truncation at the 1st and 99th centiles of the distribution across cohorts to minimise influence of extreme observations in FP modelling. 6

eFigure 2. Fitted and empirical Bayes study-specific functions for BMI-CHD association in the 10 studies with most events (top: 1369 to 5829 events, mean = 3012) as compared to the 10 studies with fewest events (bottom: 11 to 26 events, mean = 17).* Dataset 1, adjusted for confounders. Studies are indicated by same line colour within each of the top and bottom panels. Fitted study-specific hazard ratios are shown only above a lower limit of 0.25 for clarity. 7

eFigure 3. Meta-analysis of BMI-ACM association using FPs: Comparison of metacurve vs. mvmeta results with study-specific powers (SSP) or common powers (CP). Dataset 1, adjusted for confounders. With SSP, only metacurve can be used. 8

eFigure 4. Meta-analysis of BMI-ACM association using categorisation approach. Dataset 1, adjusted for confounders. Overlapping data points are slightly offset in the x-axis for better clarity. 9

eFigure 5. Comparison of metacurve vs. mvmeta results for BMI-CHD association with study-specific powers (SSP) or common powers (CP). Dataset 2, adjusted for confounders and mediators. With SSP, only metacurve can be used. 10

eFigure 6. Comparison of metacurve vs. mvmeta results for BMI-ACM association with study-specific powers (SSP) or common powers (CP). Dataset 2, adjusted for confounders and mediators. With SSP, only metacurve can be used. 11

eFigure 7. Comparison of metacurve results for BMI-ACM association when varying the centring of BMI from 20 to 35 kg/m^2. Dataset 1, adjusted for confounders. 12

eTable 1. Summary of modelling approaches, showing options in bold. All methods have study-specific parameters.

**Name**	**Choices within study**	**What is meta-analysed**	**Meta-analysis across studies**	
			**Choices**	**Meta-analysis model**
Methods compared: FP methods				
Metacurve	**Study-specific or common powers**	HRs	**Reference level x_0**	UV **CE/RE**
Mvmeta	Common powers	Coefficients	**-**	MV **CE/RE**
Alternative method: Categorised exposure				
Categorised exposure	**Number of categories**	HRs	**-**	**UV / MV CE/RE**

FP: fractional poynomial. HR: hazard ratio. UV: Univariate. MV: multivariate. CE: common-effect. RE: random-effects.

eFigure 1. Box plots of distributions of age and BMI in 121 cohorts contributing to analyses sorted in ascending order of mean age. Dataset 1. BMI data are shown with truncation at the 1st and 99th centiles of the distribution across cohorts to minimise influence of extreme observations in FP modelling.

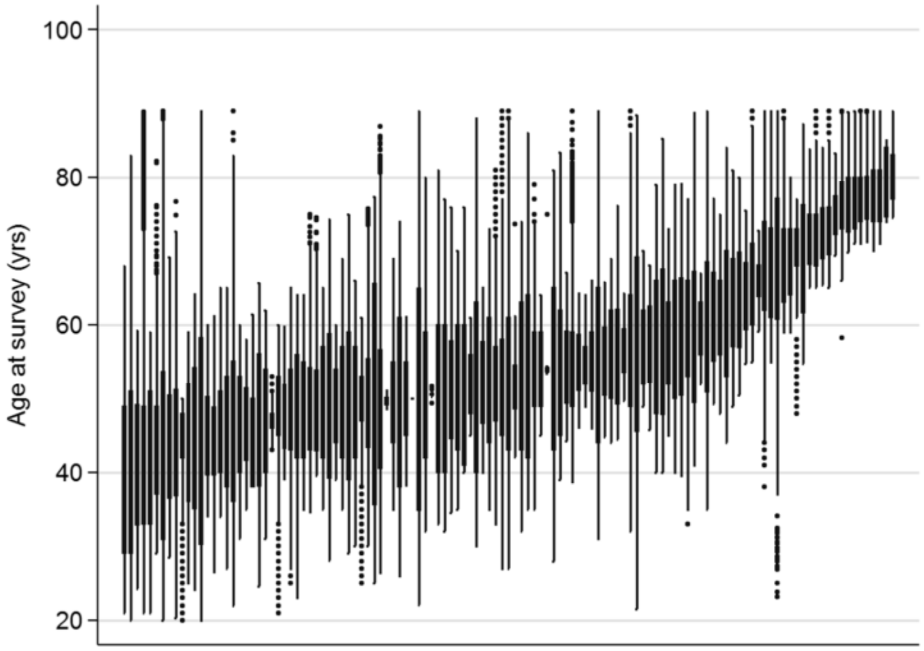

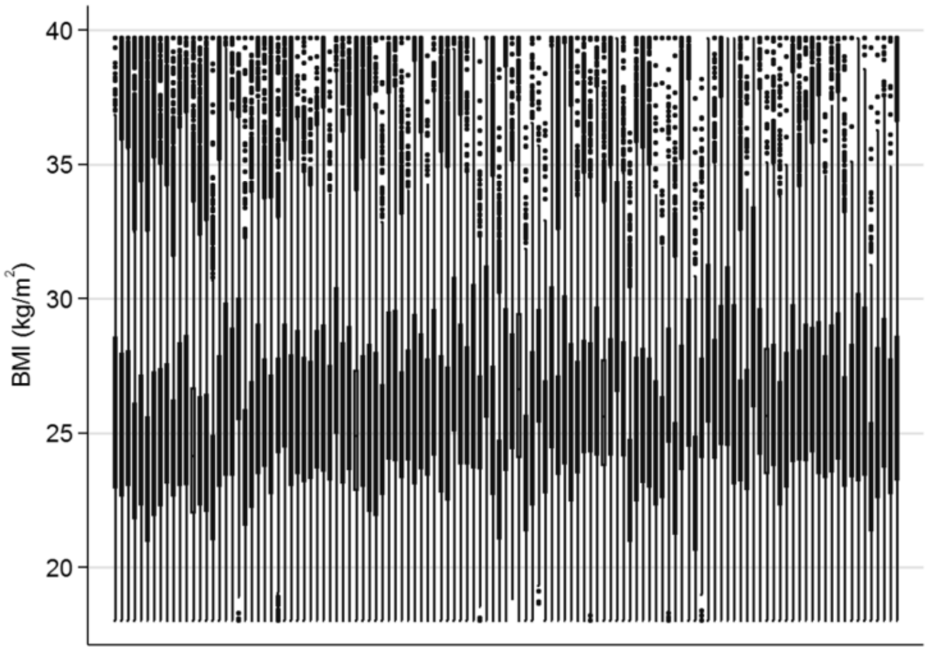

eFigure 2. Fitted and empirical Bayes study-specific functions for BMI-CHD association in the 10 studies with most events (top: 1369 to 5829 events, mean = 3012) as compared to the 10 studies with fewest events (bottom: 11 to 26 events, mean = 17). Dataset 1, adjusted for confounders. Studies are indicated by same line colour within each of the top and bottom rows. Fitted study-specific hazard ratios are shown only above a lower limit of 0.25 for clarity.

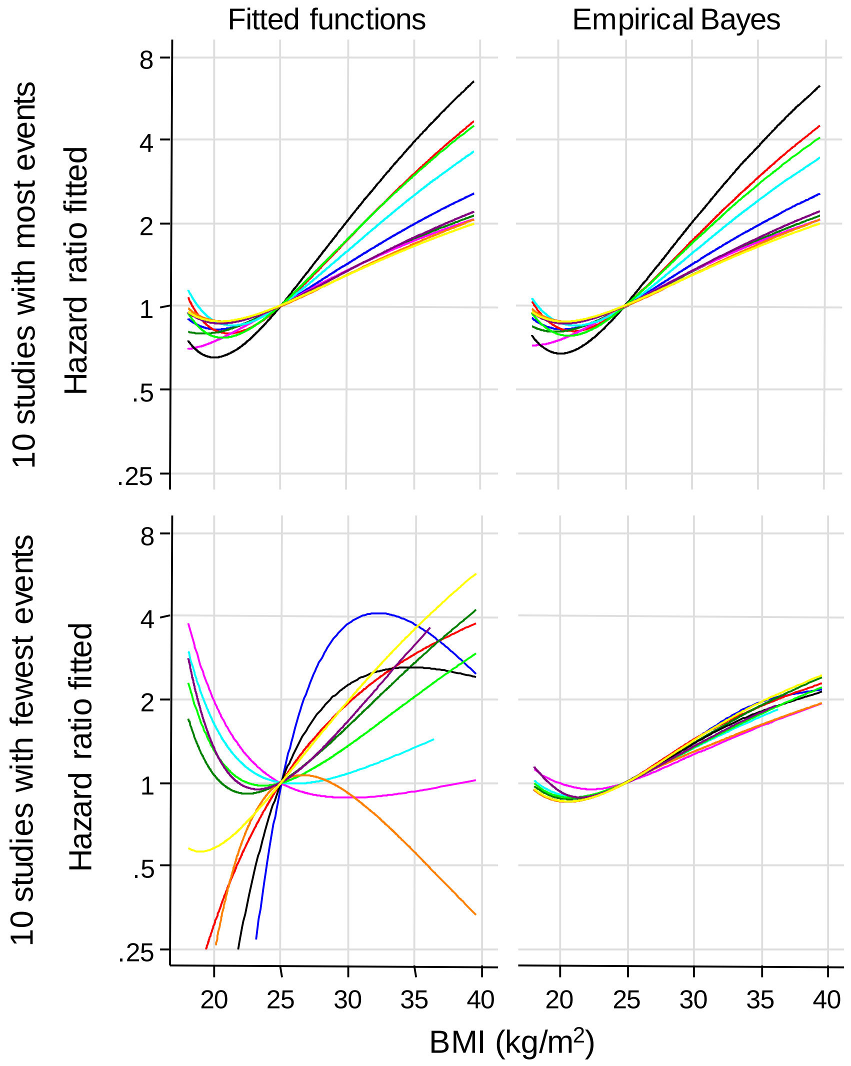

eFigure 3. Meta-analysis of BMI-ACM association using FPs: Comparison of metacurve vs. mvmeta results with study-specific powers (SSP) or common powers (CP). Dataset 1, adjusted for confounders. With SSP, only metacurve can be used.

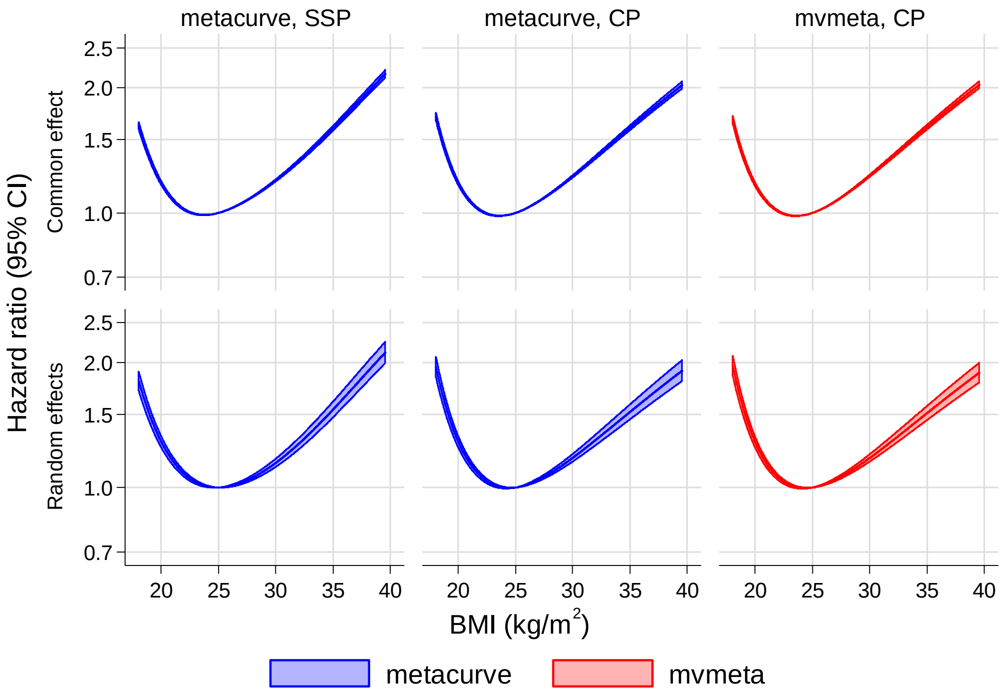

eFigure 4. Random-effects meta-analysis of BMI-ACM association using categorisation approach. Dataset 1, adjusted for confounders. Overlapping data points are slightly offset in the x-axis for better clarity.

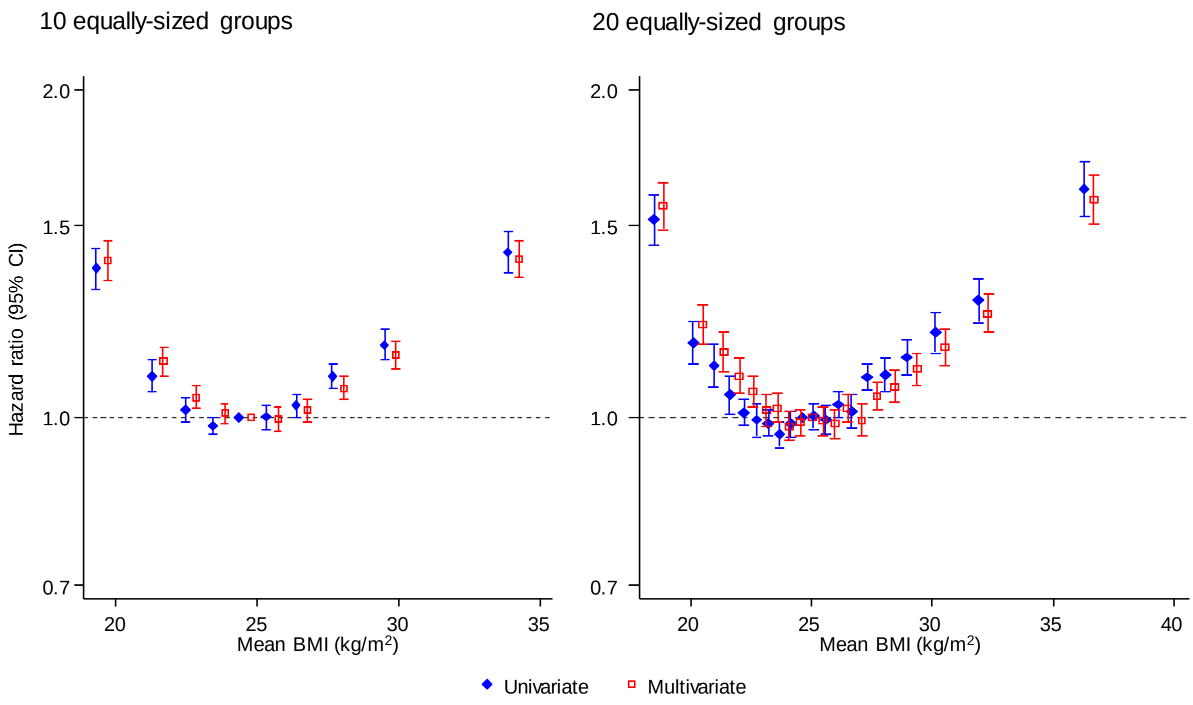

eFigure 5. Comparison of metacurve vs. mvmeta results for BMI-CHD association with study-specific powers (SSP) or common powers (CP). Dataset 2, adjusted for confounders and mediators. With SSP, only metacurve can be used.

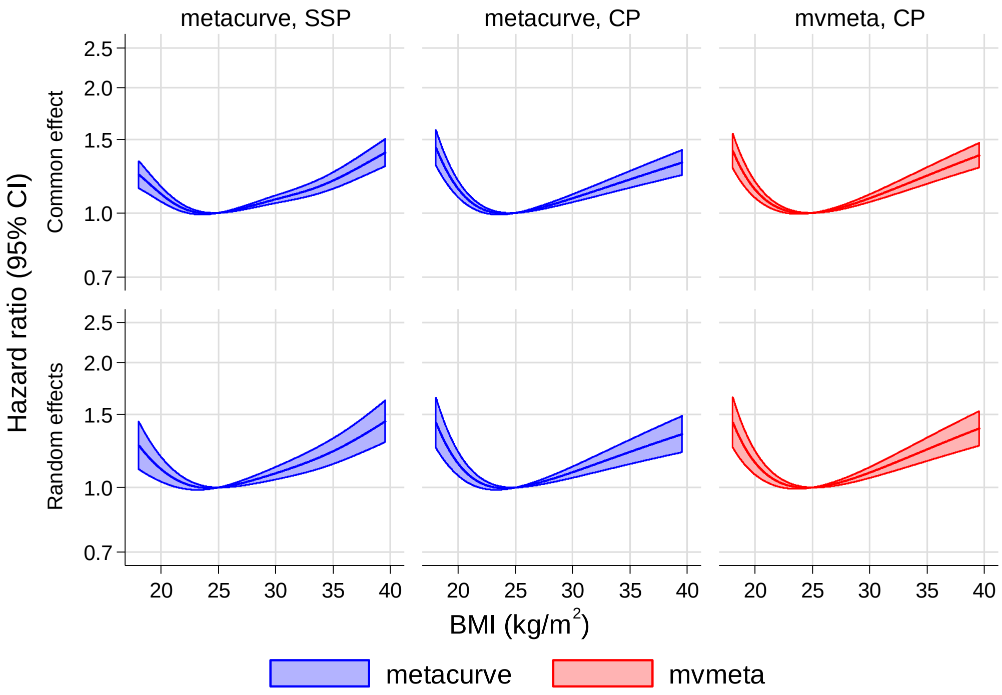

eFigure 6. Comparison of metacurve vs. mvmeta results for BMI-ACM association with study-specific powers (SSP) or common powers (CP). Dataset 2, adjusted for confounders and mediators. With SSP, only metacurve can be used.

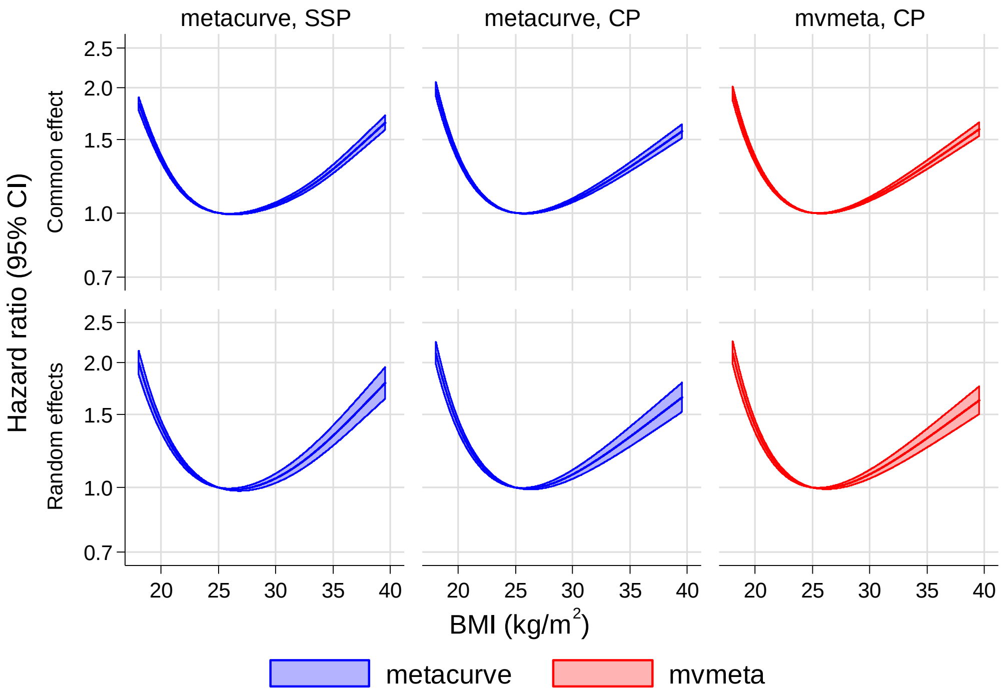

eFigure 7. Comparison of metacurve results for BMI-ACM association when varying the reference level of BMI (Ref) from 20 to 35 kg/m^2. Dataset 1, adjusted for confounders.

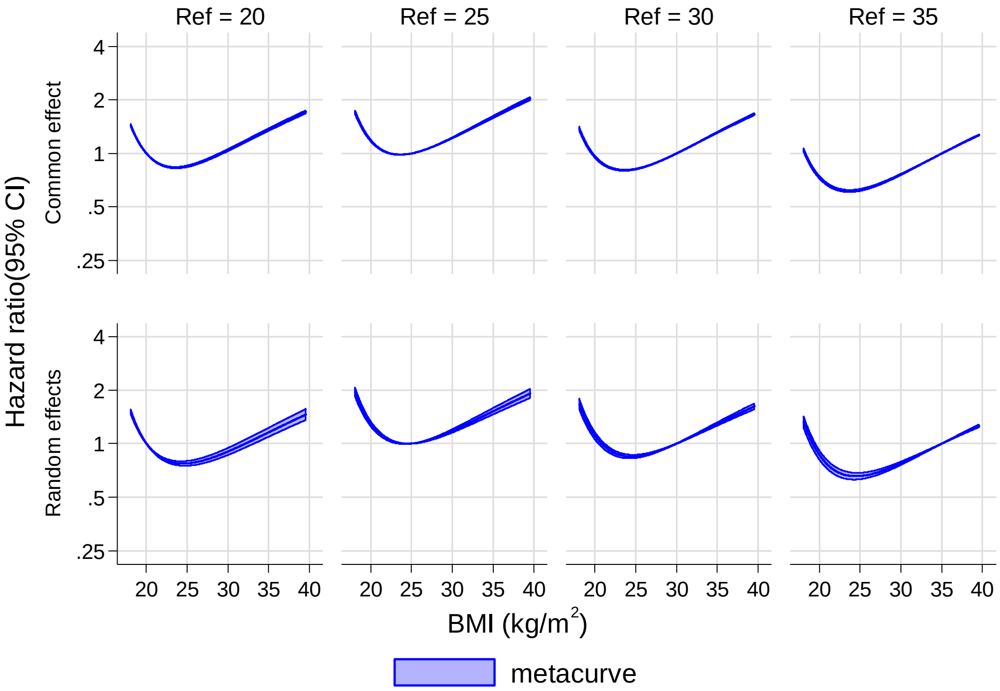

Supplement: Supplementary file 1 — SIM7974_supplementary.pdf [file SIM-38-326-s001.pdf]
